# Delayed Diagnosis of Tuberculous Coxitis in a Child Initially Misdiagnosed as Septic Arthritis: A Case Report

**DOI:** 10.1002/rcr2.70404

**Published:** 2025-11-10

**Authors:** Egi Azhar Rafsanjani, Retno Ashi Setyoningrum, Muhammad Tholhah Azam

**Affiliations:** ^1^ Department of Child Health Dr Soetomo General Academic Hospital Surabaya Indonesia; ^2^ Department of Child Health, Faculty of Medicine Universitas Airlangga Surabaya Indonesia

**Keywords:** coxitis, paediatrics, radiology, surgery, tuberculosis

## Abstract

Tuberculous coxitis is a rare form of extrapulmonary tuberculosis in children, often misdiagnosed due to its subtle presentation and resemblance to other joint disorders. We report the case of a 12‐year‐old boy with a seven‐month history of progressive right hip pain, joint deformity, and a draining sinus, initially mismanaged as a nonspecific infection. Imaging revealed destructive changes in the right hip, and molecular testing confirmed *Mycobacterium tuberculosis* from both gastric aspirate and intra‐articular pus. The patient underwent anti‐tuberculosis therapy and surgical debridement with internal fixation. Histopathology confirmed tuberculous arthritis. Significant clinical improvement was observed postoperatively, with restoration of mobility. This case underscores the importance of early suspicion of skeletal TB in chronic monoarthritis, especially in endemic regions. Delayed diagnosis may lead to joint destruction and disability, but multidisciplinary management and timely intervention can yield favorable outcomes.

## Graphical abstract

We report a paediatric case of tuberculous coxitis initially misdiagnosed as septic arthritis, resulting in delayed diagnosis and joint destruction. Molecular testing confirmed *Mycobacterium tuberculosis* , and the patient underwent anti‐TB therapy and surgical intervention. Early recognition and multidisciplinary management are essential to prevent disability in skeletal TB.

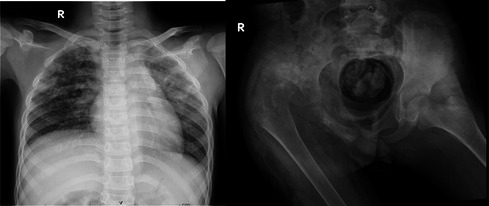

## 1 Introduction

Tuberculosis (TB) remains a significant global health burden, particularly in developing countries with high transmission rates. While pulmonary TB is the most common presentation, extrapulmonary TB (EPTB) can affect various organs, including bones and joints. Musculoskeletal TB accounts for 10%–35% of EPTB, yet tuberculous coxitis, a form affecting the hip joint, is a rare and often overlooked entity in paediatric populations [1].

Its insidious onset, nonspecific symptoms, and radiologic similarities with other joint pathologies such as septic arthritis or transient synovitis, make early diagnosis challenging. Children with TB frequently lack classic pulmonary symptoms, further complicating clinical recognition [2].

In resource‐limited settings, delayed diagnosis can lead to severe joint destruction, deformity, and long‐term disability [3]. This case report presents a rare instance of tuberculous coxitis in a child, initially misdiagnosed as septic arthritis, emphasising the need for heightened clinical suspicion and a multidisciplinary diagnostic approach in children presenting with chronic monoarthritis in endemic regions [4].

## 2 Case Report

A 12‐year‐10‐month‐old boy presented to the emergency department with a seven‐month history of right pelvic pain, progressive stiffness, and inability to walk. The symptoms began with a small lump over the right hip, which gradually enlarged and discharged purulent fluid. There was no associated fever, cough, or night sweats. No previous trauma or known contact with tuberculosis (TB) was reported. The patient experienced approximately 2 kg of weight loss over the past 6 months.

On further questioning, the patient denied a productive cough but reported chronic fatigue, decreased appetite, and persistent weight loss. Initially, he continued attending school but gradually missed classes due to worsening hip pain and restricted movement. He walked with a limp, required assistance for ambulation, and remained partially independent but not wheelchair‐bound.

Initial care at a district hospital included analgesics and antibiotics without further follow‐up. Plain radiography performed at that time showed cortical irregularity and lytic destruction over the femoral head and acetabular region, interpreted as non‐specific bone infection. However, no definitive diagnosis was made, and the possibility of tuberculosis was not considered at that stage; therefore, a supporting chest radiograph was not contemplated, contributing to the diagnostic delay. Due to persistent symptoms, the patient was referred to a tertiary care center.

The child was treated with antibiotics for presumed septic arthritis, and the clinical course closely mimicked septic arthritis and transient synovitis, delaying suspicion of tuberculosis. He had received at least two courses of broad‐spectrum antibiotics over a six‐week period without clinical improvement. On admission, the patient appeared chronically ill and undernourished, with weight and height both below the 5th percentile. Physical examination revealed a flexion contracture of the right hip and a draining sinus. Hip movement was limited and painful. There was no lymphadenopathy or distal neurovascular deficit. Vital signs were as follows: afebrile (37.5°C), respiratory rate 22/min, pulse 92/min, SpO_2_ 98% on room air. Lung auscultation revealed bilateral coarse crackles at the lower zones. Abdominal and cardiovascular examinations were unremarkable.

**FIGURE 1:**
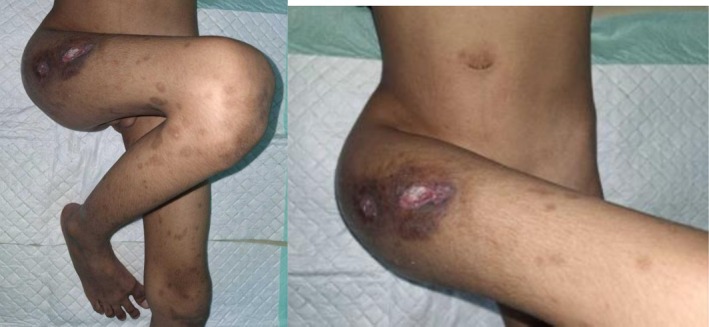
Clinical appearance on admission showing right hip contracture and active draining sinus, consistent with advanced joint infection.

The draining sinus, located over the anterolateral aspect of the right hip, measured approximately 2 cm in diameter, with thickened and indurated margins, minimal surrounding erythema, and the discharge of thick yellowish pus. The overlying skin showed hyperpigmentation but no edema. Figure 1 demonstrates the clinical appearance of the right hip, showing a draining sinus and hip deformity on admission.

**FIGURE 2:**
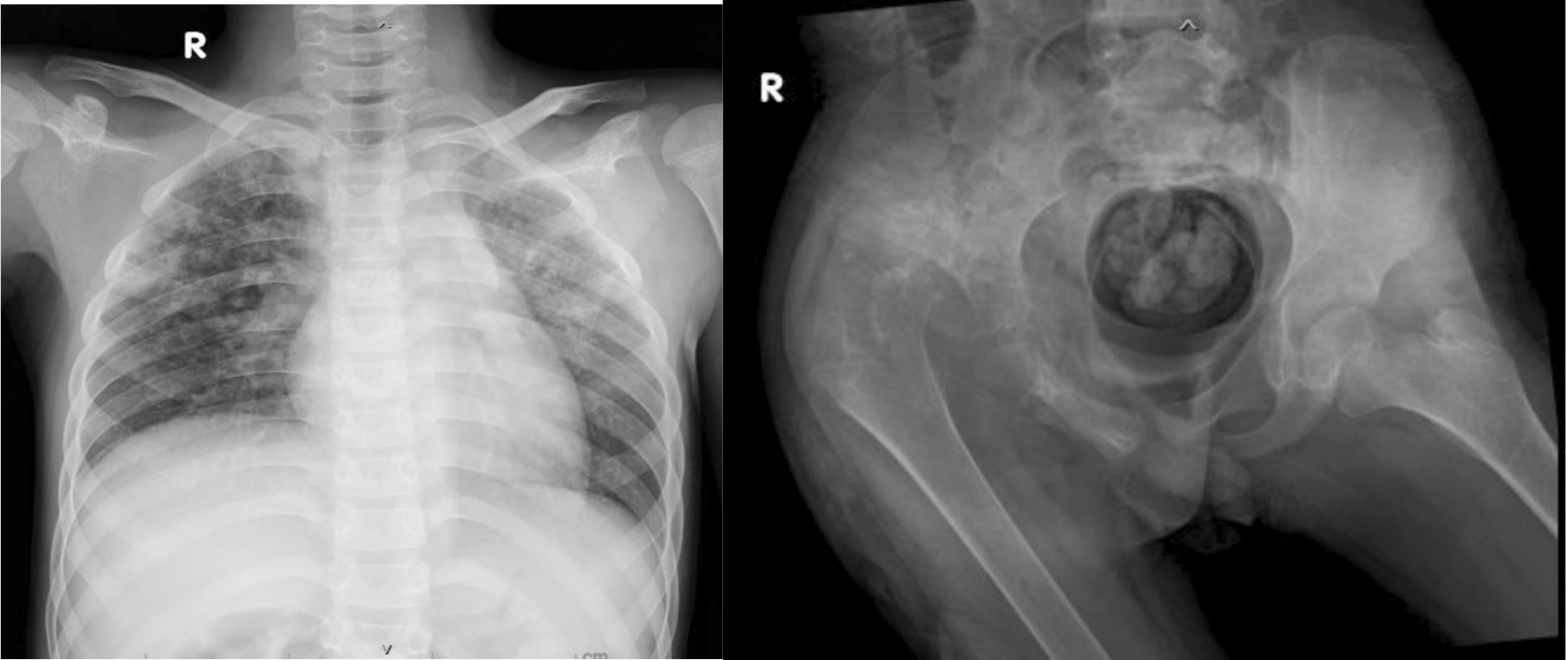
Chest and pelvic radiographs. Chest x‐ray showing bilateral patchy infiltrates in the supraparahilar regions, suggestive of pulmonary tuberculosis (Left). Pelvic radiograph showing extensive destruction of the right femoral head, neck, and acetabular roof, with posterior‐superior dislocation of the femoral head and associated soft tissue swelling, consistent with advanced tuberculous coxitis (Right).

Laboratory findings revealed leukocytosis, microcytic anaemia, and hypoalbuminemia. Specifically, haemoglobin 9.1 g/dL, total leukocyte count 13,200/μL, ESR 72 mm/h, CRP 28 mg/L, and serum albumin 3.13 g/dL. Chest radiography showed bilateral patchy infiltrates, while pelvic radiography and CT scan revealed destruction of the right femoral head and acetabular roof, dislocation of the hip joint, and associated soft tissue swelling suggestive of septic arthritis with suspected tuberculous aetiology. Figure 2 demonstrates radiographic findings of pulmonary involvement and advanced hip joint destruction associated with tuberculous coxitis. These findings were consistent with concomitant pulmonary tuberculosis, confirming dual pulmonary and skeletal involvement.

**FIGURE 3:**
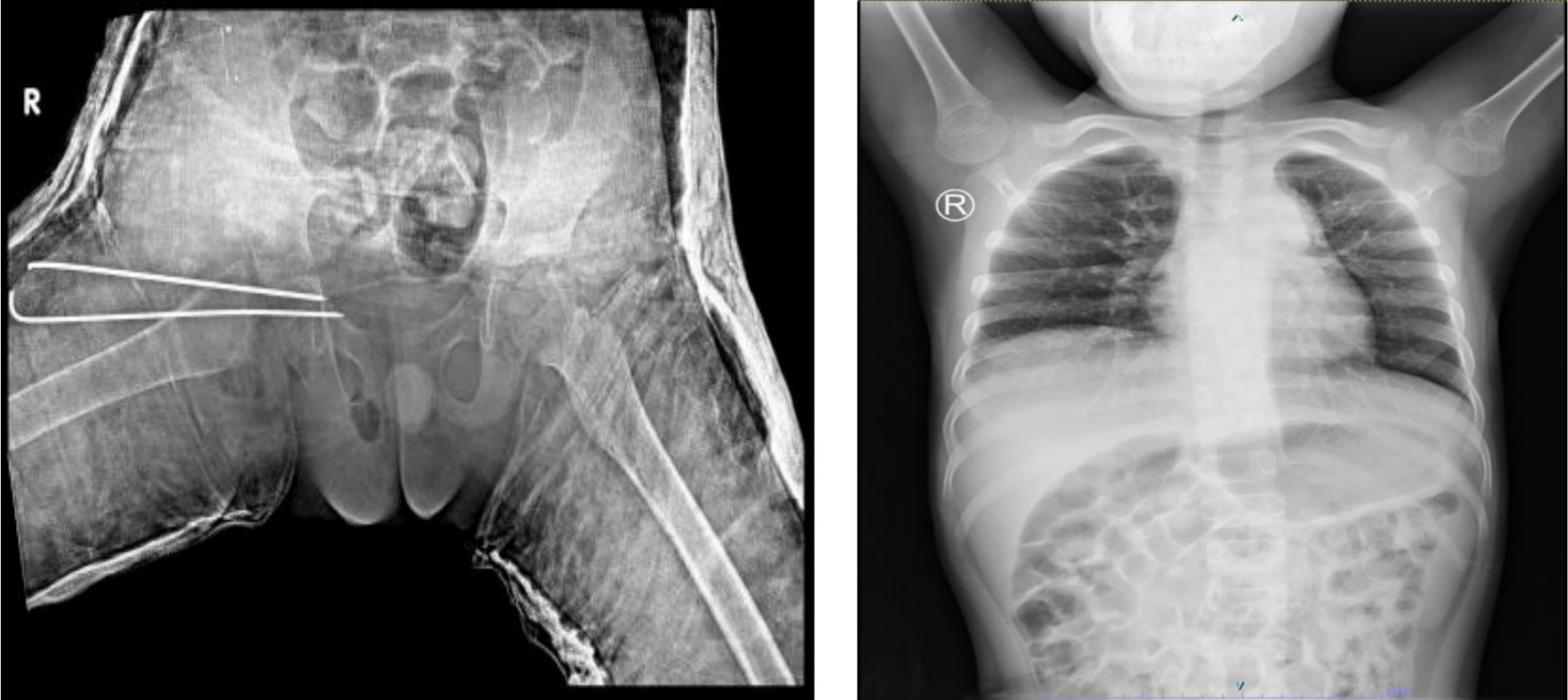
Postoperative pelvic x‐ray showing internal fixation using two smooth pins extending from the proximal right femur to the superior pubic ramus. The fixation appears stable with no evidence of loosening or malalignment (Left). Follow‐up chest radiograph obtained 2 months after initiation of anti‐tuberculosis therapy demonstrates marked resolution of bilateral patchy infiltrates, indicating early pulmonary improvement and good systemic response to treatment (Right).

A Mantoux test showed 12 mm induration, and rapid molecular testing of both gastric aspirate and intra‐articular pus detected *Mycobacterium tuberculosis* without rifampicin resistance. Subsequent microbiological confirmation from two independent specimens supported the diagnosis. *M. tuberculosis* was detected from both the gastric aspirate and hip tissue samples, with rifampicin sensitivity confirmed on GeneXpert testing performed at the Dr. Soetomo General Hospital Microbiology Laboratory (22 November and 16 December 2024, respectively). These findings confirmed drug‐sensitive *M. tuberculosis* infection involving both pulmonary and skeletal sites. The patient was initiated on standard anti‐tuberculosis therapy (2RHZE) and underwent open surgical debridement, arthrotomy, and internal fixation. Surgery was performed 5 days after the initiation of anti‐tuberculosis treatment, under appropriate infection control precautions to minimise intraoperative exposure risk for the surgical team. Histopathological analysis confirmed granulomatous inflammation with Langhans giant cells. Postoperative radiograph (Figure 3) demonstrated satisfactory alignment and fixation without evidence of loosening or hardware complications. This test was performed upon referral and not during initial district hospital workup. Surgery was performed shortly after the initiation of therapy, following infection control precautions to reduce exposure risk to OT staff. At approximately 2 months after the initiation of anti‐tuberculosis therapy, a follow‐up chest radiograph showed marked improvement of the bilateral patchy infiltrates, indicating early pulmonary recovery and good systemic response to treatment (Figure 3).

At 2 months post‐surgery, the patient had regained mobility and was ambulatory without pain. Clinical and radiological follow‐up confirmed significant improvement with no further complications. Follow‐up imaging revealed improved joint alignment and resolution of pulmonary infiltrates, indicating favourable response to combined medical and surgical therapy.

## 3 Discussion

Tuberculous coxitis is an uncommon manifestation of extrapulmonary tuberculosis (EPTB), particularly in children [1]. Its insidious onset, paucity of pulmonary symptoms, and gradual joint destruction often mimic other joint diseases such as septic arthritis or transient synovitis, leading to diagnostic delay [2]. In this case, a 12‐year‐old boy experienced progressive hip pain and stiffness for 7 months, initially treated as a nonspecific infection before referral for specialist evaluation. The absence of constitutional symptoms such as fever, night sweats, or known TB contact further contributed to the missed diagnosis. This presentation illustrates the subtle and deceptive course of paediatric osteoarticular tuberculosis and the necessity for high clinical suspicion, even when systemic features are absent [3].

Importantly, although the chest radiograph was eventually abnormal, it should ideally have been performed earlier as part of the initial diagnostic work‐up for a child with chronic joint pain. In TB‐endemic areas, performing an early chest x‐ray in patients presenting with prolonged monoarthritis can be a key step to uncover underlying pulmonary tuberculosis, which in turn may expedite diagnosis and appropriate management [4]. This case exemplifies that simple and inexpensive imaging such as chest radiography, if performed early, may provide the first diagnostic clue to systemic TB involvement, preventing further joint destruction and disability.

A review of the literature indicates that the spine is the most frequently affected site of osteoarticular tuberculosis, accounting for approximately 50% of cases, followed by the knee (20%–30%) and hip (15%–20%) [5]. Although less common than spinal involvement, tuberculous coxitis carries significant functional consequences because the hip is a major weight‐bearing joint^25^. Delayed recognition of hip tuberculosis, as seen in this case, often leads to joint deformity, limb length discrepancy, or permanent disability.

Radiologically, tuberculous coxitis typically exhibits Phemister's triad: periarticular osteoporosis, marginal erosions, and gradual joint space narrowing [4]. In our patient, pelvic imaging showed destructive changes of the femoral head and acetabulum, consistent with advanced disease. Follow‐up chest radiograph after 2 months of anti‐tuberculosis therapy demonstrated marked resolution of bilateral patchy infiltrates, indicating a favourable systemic response. Diagnostic confirmation remains challenging in such paucibacillary disease; however, molecular assays such as GeneXpert MTB/RIF have revolutionised diagnosis by enabling rapid detection of *M. tuberculosis* and rifampicin sensitivity. In this case, *M. tuberculosis* was detected from both gastric aspirate and hip tissue specimens, confirming drug‐sensitive TB involving both pulmonary and skeletal systems. Histopathological analysis revealing granulomatous inflammation with Langhans giant cells further supported the diagnosis.

Optimal management of skeletal TB involves a multidisciplinary approach combining medical therapy and surgical intervention [4]. The patient underwent open debridement and internal fixation, followed by a full course of anti‐tuberculosis chemotherapy (HRZE regimen). Two months postoperatively, he regained pain‐free ambulation, with radiological evidence of bone healing and stable fixation. Early surgical intervention complemented by anti‐TB therapy is essential to preserve joint integrity and function.

In summary, this case underscores the diagnostic challenge of tuberculous coxitis in children and emphasises the crucial role of early imaging and molecular testing in achieving timely diagnosis. The key learning point is that performing a chest radiograph in patients presenting with chronic joint pain may help uncover underlying tuberculosis and guide earlier diagnosis and management. In TB‐endemic settings, clinicians must maintain a high index of suspicion for skeletal TB, even in the absence of respiratory symptoms, to prevent irreversible joint damage and lifelong disability.

## Ethics Statement

Egi Azhar Rafsanjani was responsible for the clinical management of the patient and drafted the initial manuscript. Retno Ashi Setyoningrum supervised the diagnostic and therapeutic approach and contributed to the critical revision of the manuscript. Muhammad Tholhah Azam provided radiological and surgical input and participated in manuscript editing and final approval. All authors reviewed and approved the final version of the manuscript. All authors are affiliated with the same institution and contributed equally to this work.

## Consent

The authors declare that written informed consent was obtained for the publication of this manuscript and accompanying images using the form provided by the Journal.

## Conflicts of Interest

The authors declare no conflicts of interest.

## Data Availability

The data that support the findings of this study are available from the corresponding author upon reasonable request.
